# A randomized controlled trial of lusutrombopag in Japanese patients with chronic liver disease undergoing radiofrequency ablation

**DOI:** 10.1007/s00535-018-1499-2

**Published:** 2018-08-13

**Authors:** Ryosuke Tateishi, Masataka Seike, Masatoshi Kudo, Hideyuki Tamai, Seiji Kawazoe, Takayuki Katsube, Toshimitsu Ochiai, Takahiro Fukuhara, Takeshi Kano, Katsuaki Tanaka, Mineo Kurokawa, Kazuhide Yamamoto, Yukio Osaki, Namiki Izumi, Michio Imawari

**Affiliations:** 1 Department of Gastroenterology, The University of Tokyo Hospital, 7-3-1 Hongo, Bunkyo-ku, Tokyo, 113-8655 Japan; 2 Department of Gastroenterology, Oita University Hospital, 1-1 Hasamamachi-idaigaoka, Yufu, 879-5593 Japan; 3 Department of Gastroenterology and Hepatology, Kindai University Faculty of Medicine, 377-2 Ono-higashi, Osaka-sayama, 589-8511 Japan; 4 Second Department of Internal Medicine, Wakayama Medical University Hospital, 811-1 Kimiidera, Wakayama, 641-0012 Japan; 5 Department of Hepatobiliary and Pancreatology, Saga-ken Medical Centre Koseikan, 1-12-9 Mizugae, Saga, 840-0054 Japan; 6 Shionogi and Co., Ltd., 1-4, Shibata 1-chome, Kita-ku, Osaka, 530-0012 Japan; 7 Yokohama City University Medical Center, Gastroenterological Center, 4-57 Urafune, Minami-ku, Yokohama, 232-0024 Japan; 8 Department of Hematology and Oncology, The University of Tokyo Hospital, 7-3-1 Hongo, Bunkyo-ku, Tokyo, 113-8655 Japan; 9 Gastroenterology and Hepatology Department, Okayama University Hospital, 2-5-1 Shikata-cho, Kita-ku, Okayama, 700-8558 Japan; 10 Department of Gastroenterology and Hepatology, Japanese Red Cross Society Osaka Hospital, 5-30 Fudegasakicho, Tennoji-ku, Osaka, 543-8555 Japan; 11 Japanese Red Cross Society Musashino Hospital, 1-26-1 Kyonancho, Musashino, Tokyo, 180-8610 Japan; 12 Institute for Gastrointestinal and Liver Diseases, Shin-yurigaoka General Hospital, 255 Furusawa, Asao-ku, Kawasaki, 215-0026 Japan; 13 Present Address: Department of Hepatology, Wakayama Rousai Hospital, 93-1 Kinomoto, Wakayama, 640-8505 Japan; 14 Present Address: Kawazoe Medical Clinic, 1834-1 Fukuyoshi, Shiroishi-cho, Kishima-gun, Saga, 849-1113 Japan; 15 Present Address: Japanese Red Cross Society Hadano Hospital, 1-1 Tatenodai, Hadano, 257-0017 Japan; 16 Present Address: Okayama Saiseikai General Hospital, 2-25 Kokutaicho, Kita-ku, Okayama, 700-8511 Japan; 17 Present Address: Meiwa Hospital, 4-31 Agenaruocho, Nishinomiya, 663-8186 Japan

**Keywords:** Chronic liver disease, Lusutrombopag, Radiofrequency ablation, Thrombocytopenia, Thrombopoietin receptor agonist

## Abstract

**Background:**

Thrombocytopenia represents an obstacle for invasive procedures in chronic liver disease (CLD) patients. We aimed to estimate the appropriate dose and evaluate the efficacy and safety of lusutrombopag for the treatment of thrombocytopenia before percutaneous liver radiofrequency ablation (RFA) for primary hepatic cancer in patients with CLD.

**Methods:**

In this multicenter, randomized, double-blind, placebo-controlled study conducted in Japan, 61 CLD patients with platelet count < 50 × 10^3^/µL at screening were randomized to placebo or lusutrombopag 2, 3, or 4 mg once daily for 7 days, followed by a 28-day post-treatment assessment period. The primary efficacy endpoint was the proportion of patients who did not require platelet transfusion before RFA. The pre-specified key secondary efficacy endpoint was the proportion of responders. Adverse events (AEs) and thrombosis-related AEs were evaluated.

**Results:**

The proportion of patients who did not require platelet transfusion before RFA and that of responders were significantly higher (*p* < 0.01) in the 2-mg (80.0, 66.7%), 3-mg (81.3, 68.8%), and 4-mg groups (93.3, 80.0%) compared with the placebo group (20.0, 6.7%) and showed a dose-dependent effect. The incidence of AEs was 97.8 and 100% in the lusutrombopag (all groups) and placebo groups, respectively; no dose-related increase was observed. Four patients experienced thrombosis-related events (one each in the placebo and 2-mg groups, and two in the 4-mg group). A total of 16 (18%) adverse drug reactions occurred in the safety analysis set.

**Conclusions:**

Lusutrombopag 3 mg once daily for 7 days was effective without raising concerns about excessive increases in platelet count.

**Clinical trial registration:**

The study is registered at JapicCTI-121944.

**Electronic supplementary material:**

The online version of this article (10.1007/s00535-018-1499-2) contains supplementary material, which is available to authorized users.

## Introduction

In patients with chronic liver disease (CLD), thrombocytopenia is a very common complication, with levels below the normal range being reported in up to 76% of patients with cirrhosis [1, 2]. In patients with severe thrombocytopenia and levels below 50 × 10^3^/µL, thrombocytopenia represents an obstacle for invasive diagnostic or therapeutic procedures [1, 3]. Multiple factors are thought to contribute to the development of thrombocytopenia in CLD patients. Decreased levels/activity of thrombopoietin (TPO), use of chemotherapy for hepatic cancers, bone marrow inhibition by excessive alcohol ingestion, hypersplenism, antiplatelet antibodies, and antiviral treatment-induced myelosuppression may all contribute to the development of thrombocytopenia in CLD [2].

In patients with severe thrombocytopenia who require invasive procedures, the only current nonsurgical treatment option available is the use of platelet transfusions to reduce the risk of hemorrhagic events during and after the procedures [2]. However, there are several limitations and potential complications associated with platelet transfusions, including febrile nonhemolytic and allergic reactions, risk of infection and platelet refractoriness, need for hospitalization, and high cost [4–7]. Alternatives that are more permanent and less common than repeat of platelet transfusions for patients with CLD and severe resistant thrombocytopenia are procedures such as splenectomy or splenic artery embolization [2, 3, 8, 9]. However, concerns remain regarding the long-term outcomes of splenectomy and its impact on immunological function as well as the morbidity associated with procedure-related complications [10].

Lusutrombopag is a chemically synthesized, orally active small-molecule human TPO receptor agonist (TPO-RA) discovered and developed by Shionogi & Co., Ltd (Osaka, Japan). Lusutrombopag acts on the transmembrane domain of human TPO receptors, activates the signal transduction pathway in the same fashion as endogenous TPO, and induces platelet production [11]. Phase 1 studies indicate that lusutrombopag induces thrombopoiesis with once-daily oral administration in humans, and the pharmacokinetics of lusutrombopag are similar between Japanese and Caucasian healthy subjects [12].

The primary objective of this study was to investigate the dose of lusutrombopag required to reduce the need for pretreatment platelet transfusions associated with percutaneous liver radiofrequency ablation (RFA) for primary hepatic carcinoma in Japanese CLD patients with thrombocytopenia. The secondary objectives of this study were to compare the efficacy and safety profiles of lusutrombopag with those of placebo, and to evaluate the pharmacokinetic properties of lusutrombopag.

## Methods

### Study design and treatment

This was a multicenter, randomized, double-blind, parallel-group, placebo-controlled, phase 2b dose-finding study conducted from August 2012 to April 2013 in 63 centers (Supplement S1) in Japan. Written informed consent was obtained from each patient included in the study, and the study protocol conforms to the ethical guidelines of the 1975 Declaration of Helsinki as reflected in a priori approval by the institution’s human research committee. The study was conducted in compliance with the Ordinance on the Standards for the Conduct of Clinical Trials on Drugs and Good Clinical Practice guidelines and was registered at www.clinicaltrials.jp (JapicCTI-121944).

**Fig. 1:**
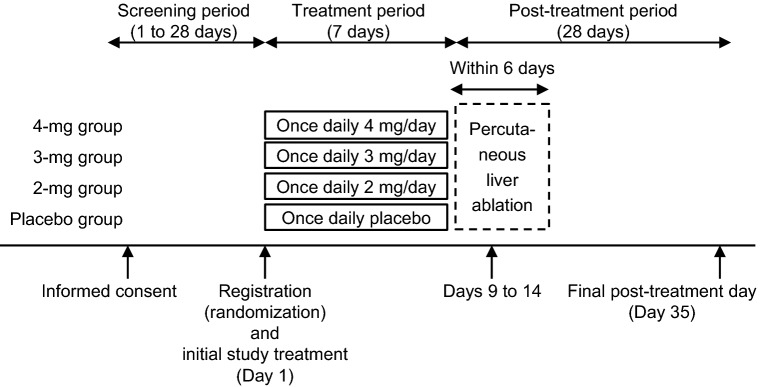
Study design

The study consisted of three study periods: the screening period (1–28 days), the treatment period (7 days), and the post-treatment period (28 days) (Fig. 1). Potential patients who provided written informed consent were screened to assess their eligibility in the screening period. Eligible patients were randomized in a 1:1:1:1 ratio using a stochastic minimization method to one of the four following groups: placebo; or 2, 3, or 4 mg lusutrombopag QD. Child–Pugh class (Child–Pugh A or B) and platelet count (< 35 × 10^3^/µL,  35 × 10^3^/µL to < 45 × 10^3^/µL, or ≥ 45 × 10^3^/µL) at screening were used as randomization factors. The random allocation of patients who were enrolled by investigators was implemented centrally and conveyed to investigators by fax. The doses were determined based on the results of previous studies in Japanese patients scheduled for RFA (JapicCTI-101377, CTI-111625).

To prevent an excessive increase in platelet count during the course of the study, the study drug was discontinued if the platelet count was ≥ 50 × 10^3^/µL with an increase of ≥ 20 × 10^3^/µL from baseline. This was assessed on Days 5, 6, and 7 before dosing. Although the drug was discontinued in these patients, the patients continued to be evaluated in this study up to the end of the post-treatment period.

After the 7-day treatment period, patients underwent specified post-treatment study assessments (post-treatment period). RFA was allowed between Days 9 and 14. The need for platelet transfusion was determined by a platelet count < 50 × 10^3^/µL measured at one point between the completion of assessments on Day 8 and immediately before the RFA. If the platelet count was < 50 × 10^3^/µL, platelet concentrate was transfused to the patient; the amount of units was directed by the investigator.

Prohibited therapies that could interfere with assessment of efficacy or safety were excluded and are listed in Supplement S2.

### Participants

The inclusion criteria were as follows: men or women aged 20 years or older at the time of providing informed consent, patients with thrombocytopenia due to CLD, platelet count of < 50 × 10^3^/µL at screening, patients who were undergoing RFA for primary hepatic carcinoma, patients who had an Eastern Cooperative Oncology Group performance status grade 0 or 1, patients who were able to remain hospitalized between 5 and 14 days after the initiation of the study treatment, and patients who agreed to use an appropriate method of contraception during the study.

The exclusion criteria were as follows: patients with any other causes of thrombocytopenia; patients with a history of liver transplantation; patients with Child–Pugh class C liver disease, uncontrollable hepatic encephalopathy or uncontrollable ascites; patients with active malignant tumor other than primary hepatic cancer; patients who had undergone splenectomy; patients with a history of portal vein thrombosis (PVT); and patients for whom hepatopetal portal blood flow could not be demonstrated using Doppler ultrasonography.

### Assessments

The primary efficacy endpoint was the proportion of patients who did not require platelet transfusion before RFA. The secondary efficacy endpoints were: (a) frequency of platelet transfusion and dose (units) of platelets transfused during the study; (b) the proportion of responders (those with platelet count ≥ 50 × 10^3^/µL with an increase of ≥ 20 × 10^3^/µL from baseline), which was analyzed excluding platelet counts after the first platelet transfusion; (c) the duration of sustained platelet count increase (number of days during which platelet count was maintained as ≥ 50 × 10^3^/µL, ≥ 70 × 10^3^/µL, or met the criteria for responder) and comparison between each group of the lusutrombopag doses without platelet transfusion and placebo group with platelet transfusion; and (d) the time course change in platelet count. The pharmacokinetics of lusutrombopag at steady state was assessed based on plasma lusutrombopag concentrations. The elimination half-life (*t*_1/2,z_) was calculated based on plasma concentrations after the last dose.

For safety assessments, adverse events (AEs), thrombosis-related AEs/assessment of PVT, and bleeding-related AEs were evaluated. Thromboembolic events were proactively identified by imaging (computed tomography or magnetic resonance imaging) in the screening period and between 3 and 10 days after RFA, to examine for any possible thrombosis in the portal vein or mesenteric vein. Furthermore, pro- and anti-coagulant factors [i.e., von Willebrand factor activity, antithrombin III (%), protein C activity, and free protein S antigen] were assessed to detect thrombotic condition at screening.

AEs were defined as any medical occurrence in a patient administered the study drug during the clinical trial. The severity of an AE was graded according to the Common Terminology Criteria for Adverse Events V4.0 as grade 1 (mild; a minor symptom that does not interfere with usual daily activities), grade 2 (moderate; an event that causes interference with usual daily activities or affects the clinical status), or grade 3 (severe; an event that causes interruption of the patient’s usual daily activities or has a clinically significant effect). Adverse drug reactions (ADRs) were defined as any treatment-emergent AE considered as possibly, probably, or definitely related to the study drug. Serious AEs were defined as any AE that resulted in death, a life-threatening condition, hospitalization or prolongation of existing hospitalization, persistent or significant disability/incapacity, or other medically important conditions.

### Statistical analysis

The required sample size was calculated based on the pairwise comparison of the primary endpoint between the placebo group and each lusutrombopag group without multiplicity adjustment. The treatment effects in the placebo and lusutrombopag groups were estimated at 15 and 70%, respectively, based on previous studies (JapicCTI-101377, CTI-111625). A sample size of 60 patients (15 patients per treatment group) was required to have at least 80% power at a two-sided significance level of 0.05.

The full analysis set (FAS) was the primary population for efficacy analyses and consisted of all randomized patients who received at least one dose of the study drug and had a baseline and at least one post-baseline platelet count. The safety analysis population was used for the safety analyses and was defined as all randomized patients who were administered the study drug at least once.

Summary statistics including the number of patients, means, and standard deviations (SDs) were calculated for continuous values, and the number and proportion of patients in each category were calculated. The proportion of patients who did not require platelet transfusion before RFA in each lusutrombopag group was compared with that of the placebo group by the Cochran–Mantel–Haenszel test stratified by randomization factors. The proportions of responders were also compared in a similar manner. An analysis of covariance was used to compare the durations of sustained platelet count increase between the non-recipients of platelet transfusion in each lusutrombopag group and the recipients in the placebo group. The model included the group according to the combination of treatment and platelet transfusion as a fixed effect, as well as Child–Pugh class, baseline platelet count, and the duration of the observation of platelet counts as covariates. A *p* value < 0.05 was considered statistically significant. All statistical analyses were performed using SAS (Version 9.2, SAS Institute, Cary, NC, USA) and/or Phoenix WinNonlin (Version 6.2.1, Certara USA, Inc, Princeton, New Jersey, USA). The AEs were coded based on the MedDRA (Ver. 15.1) terms, and tabulated for each treatment group by Preferred Term.

## Results

Patient disposition is shown in Supplement S3. A total of 61 patients were enrolled in the study: 15 patients in the placebo group and 15, 16, and 15 patients in the 2-, 3-, and 4-mg groups, respectively, which composed the FAS. One patient in the 2-mg group died during the post-treatment period (after completing 7 days of treatment) because of an AE (upper gastrointestinal hemorrhage). Of the 60 patients who completed the study until the end of the post-treatment period, 11 patients (three patients in the 2-mg group, three patients in the 3-mg group, and five patients in the 4-mg group) met the criterion for stopping treatment, and administration of the study drug was discontinued on subsequent days. One patient in the 3-mg group discontinued the study drug at his/her own request. Thus, 33 patients received lusutrombopag for all of 7 days.

The demographic and clinical characteristics of the patients at baseline are shown in Table 1. The baseline characteristics were well balanced across the treatment groups. The overall mean age (minimum to maximum) was 67.2 (49–85) years; 57.4% of the patients were male; and 59.0 and 41.0% were classified as Child–Pugh A and B, respectively. Most patients had chronic hepatitis C, followed by chronic hepatitis B, alcoholic liver disease, and nonalcoholic steatohepatitis. The mean ± SD platelet count was 41.0 ± 8.8 × 10^3^/μL at baseline.

**Table 1:** Demographic and clinical characteristics of the patients at baseline

	Lusutrombopag			Placebo *n* = 15
	2 mg *n* = 15	3 mg *n* = 16	4 mg *n* = 15	
Sex				
Male	7 (46.7)	9 (56.3)	11 (73.3)	8 (53.3)
Female	8 (53.3)	7 (43.8)	4 (26.7)	7 (46.7)
Age	66.0 ± 7.8	66.8 ± 8.1	65.1 ± 10.2	70.9 ± 8.6
Type of hepatitis				
CHB	4 (26.7)	3 (18.8)	2 (13.3)	1 (6.7)
CHC	10 (66.7)	11 (68.8)	12 (80.0)	12 (80.0)
ALD	3 (20.0)	2 (12.5)	1 (6.7)	1 (6.7)
NASH	0	0	0	1 (6.7)
Child–Pugh class				
A	9 (60.0)	9 (56.3)	9 (60.0)	9 (60.0)
B	6 (40.0)	7 (43.8)	6 (40.0)	6 (40.0)
Baseline platelet count, × 10^3^/µL	40.2 ± 6.4	41.8 ± 13.2	40.0 ± 7.8	41.8 ± 6.1
> 35	3 (20.0)	3 (18.8)	3 (20.0)	4 (26.7)
35 to < 45	8 (53.3)	6 (37.5)	7 (46.7)	4 (26.7)
≥ 45	4 (26.7)	7 (43.8)	5 (33.3)	7 (46.7)
Thrombopoietin (fmol/mL)	1.075 ± 0.976	1.011 ± 0.613	0.827 ± 0.581	1.722 ± 3.406
Blood coagulation/fibrinolysis parameters				
PT INR	1.31 ± 0.11	1.29 ± 0.13	1.23 ± 0.10	1.27 ± 0.10
Activated partial thromboplastin time (s)	34.61 ± 3.75	33.38 ± 4.21	34.52 ± 4.45	34.16 ± 3.78
Fibrinogen (mg/dL)	165.5 ± 38.0	187.8 ± 71.1	163.4 ± 37.2	185.8 ± 45.4
Fibrinogen degradation product (μg/mL)	3.1 ± 1.9	2.9 ± 1.6	2.8 ± 1.5	3.7 ± 3.4
Antithrombin III (%)	59.8 ± 15.6	62.2 ± 21.5	56.1 ± 14.2	60.5 ± 22.7
d-dimer (μg/mL)	0.865 ± 0.832	0.751 ± 0.698	0.577 ± 0.603	1.083 ± 1.327
Protein C activity (%)	50.1 ± 17.3	57.6 ± 25.2	49.1 ± 18.1	50.9 ± 12.8
Free protein S antigen (%)	66.4 ± 11.5	66.6 ± 15.3	65.3 ± 9.0	71.4 ± 18.4
von Willebrand factor activity (%)	230.9 ± 82.4	247.1 ± 79.2	227.3 ± 78.9	240.4 ± 94.6

Data are presented as *n* (%) or mean ± SD

*ALD* alcoholic liver disease, *CHB* chronic hepatitis B, *CHC* chronic hepatitis C, *NASH* nonalcoholic steatohepatitis, *PT INR* international normalized ratio of prothrombin time

### Efficacy

**Fig. 2:**
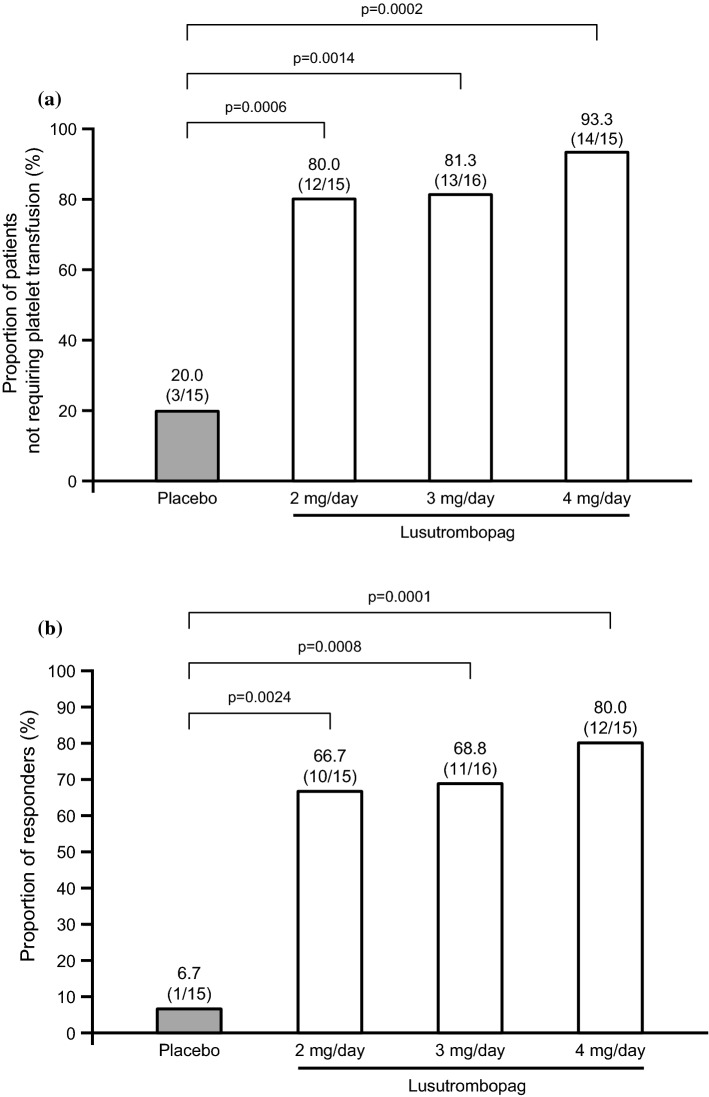
**a** Proportion of patients not requiring platelet transfusion before radiofrequency ablation and **b** responder rate. *p* values are vs placebo (Cochran–Mantel–Haenszel test)

The proportion of patients without platelet transfusion prior to the RFA, which was the primary efficacy endpoint, was significantly higher in the 2-mg group (80.0%, *p* = 0.0006), 3-mg group (81.3%, *p* = 0.0014), and 4-mg group (93.3%, *p* = 0.0002) than in the placebo group (20.0%) (Fig. 2a) and showed a modest dose-dependent effect. Similarly, the proportion of responders (secondary efficacy endpoint) during the study was significantly higher in each lusutrombopag group than the placebo group (Fig. 2b).

The number of days (adjusted mean ± SE) during which the platelet count was ≥ 50 × 10^3^/µL was significantly higher in each lusutrombopag group without platelet transfusion than in the placebo group with platelet transfusion (21.22 ± 1.56, 21.76 ± 1.66, 24.23 ± 1.67, and 4.33 ± 1.57 days in the 2-, 3-, 4-mg, and placebo groups, respectively; *p* < 0.0001 for each lusutrombopag group vs placebo). A similar result was also obtained with the platelet count meeting the criterion for responder (8.36 ± 1.73, 11.59 ± 1.84, 13.88 ± 1.85, and 0.63 ± 1.74 days in the 2-, 3-, 4-mg, and placebo groups, respectively; *p* = 0.0031, 0.0001, and < 0.0001 for each lusutrombopag group vs placebo) and with the platelet count ≥ 70 × 10^3^/µL (4.28 ± 1.59, 7.74 ± 1.68, 11.45 ± 1.69, and 0.69 ± 1.59 days in the 2-, 3-, 4-mg, and placebo groups, respectively; *p* = 0.1175, 0.0043, and < 0.0001 for each lusutrombopag group vs placebo).

**Fig. 3:**
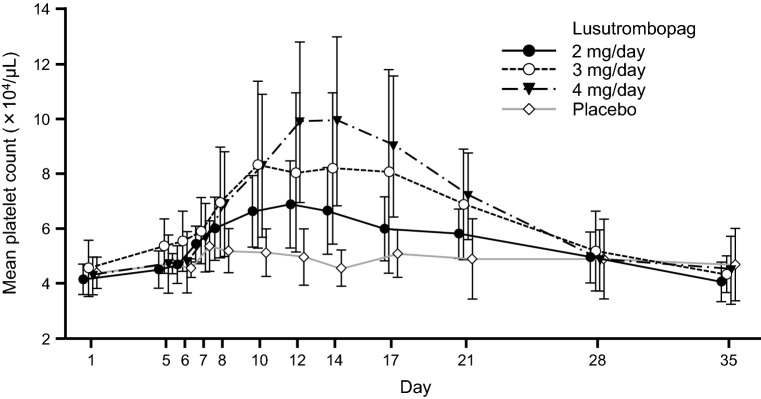
Time course of platelet count of patients without platelet transfusion. Error bars indicate standard deviation

The mean maximum platelet count in patients who did not require platelet transfusion was 74 × 10^3^/µL, 95 × 10^3^/µL, and 104 × 10^3^/µL in the 2-, 3-, and 4-mg groups, respectively; the values increased with increasing doses of lusutrombopag (Fig. 3). The mean time to reach the maximum platelet counts was 13.2–13.5 days. The maximum platelet counts in individuals in the 2-, 3-, and 4-mg groups were 93 × 10^3^/µL, 195 × 10^3^/µL, and 134 × 10^3^/µL, respectively. The mean platelet count was found to return to the baseline value in 28–35 days.

The reasons for platelet transfusion, frequency of transfusion, and mean dose of each transfusion are shown in Supplement S4. The reasons for platelet transfusion in 22 patients who received platelet transfusion during the study were “platelet count < 50 × 10^3^/µL before RFA” in 20 patients, “adverse events related to bleeding” in one patient, and “other” in four patients. The mean dose of platelet transfusion was 12.4 ± 4.1 JP units (1 JP unit contains ~ 2 × 10^10^ platelets).

### Pharmacokinetics

**Fig. 4:**
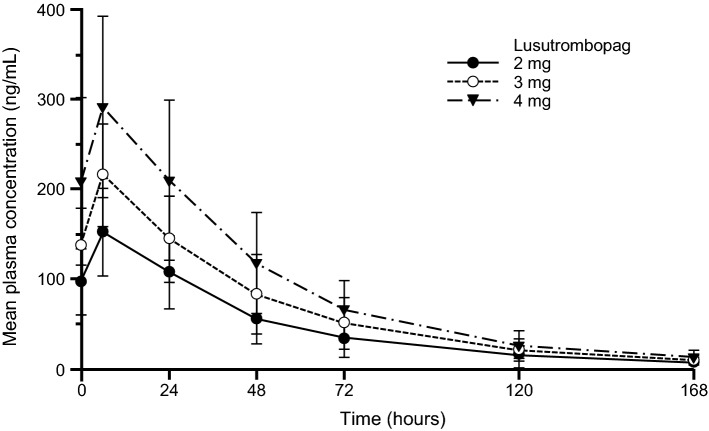
Plasma concentration of lusutrombopag. Error bars indicate standard deviation

The plasma lusutrombopag concentration after the last dose increased in a dose-proportional manner (Fig. 4). The geometric mean of elimination half-life was relatively constant regardless of the lusutrombopag dose (35.5, 38.3, and 36.5 h in the 2-, 3-, and 4-mg groups, respectively).

### Safety

There were no AEs leading to study drug discontinuation. A total of 469 AEs were reported in 60 of 61 patients (98.4%) (Table 2). Frequent AEs (incidence ≥ 20% in any lusutrombopag group and at a rate at least twice compared with placebo) were increased serum aspartate transaminase, alanine transaminase, and blood bilirubin and postprocedural hemorrhage. No dose-related increase in the incidence of AEs was observed.

**Table 2:** Incidence of adverse events

	Lusutrombopag			Placebo *n* = 15
	2 mg *n* = 15	3 mg *n* = 16	4 mg *n* = 15	
Patients with AE	15	16	14	15
No. of AEs	122	115	105	127
Incidence of AEs	100%	100%	93.3%	100%
Frequent AEs^a,b^				
Constipation	3 (3) 20.0%	2 (2) 12.5%	1 (1) 6.7%	3 (3) 20.0%
Postoperative fever	10 (10) 66.7%	9 (9) 56.3%	7 (7) 46.7%	6 (7) 40.0%
Procedural hypertension	10 (13) 66.7%	8 (9) 50.0%	6 (7) 40.0%	8 (9) 53.3%
Procedural pain	8 (8) 53.3%	8 (10) 50.0%	9 (10) 60.0%	7 (8) 46.7%
Postprocedural hemorrhage	2 (2) 13.3%	0	3 (3) 20.0%	1 (1) 6.7%
Procedural nausea	1 (1) 6.7%	0	3 (3) 20.0%	2 (2) 13.3%
AST increased	10 (12) 66.7%	10 (10) 62.5%	9 (9) 60.0%	3 (3) 20.0%
ALT increased	8 (8) 53.3%	6 (6) 37.5%	5 (5) 33.3%	0
Oxygen saturation decreased	4 (5) 26.7%	6 (7) 37.5%	5 (6) 33.3%	4 (4) 26.7%
Fibrin D-dimer increased	3 (3) 20.0%	5 (5) 31.3%	3 (3) 20.0%	5 (5) 33.3%
Fibrin degradation products increased	2 (2) 13.3%	5 (5) 31.3%	1 (1) 6.7%	4 (4) 26.7%
Blood bilirubin increased	4 (4) 26.7%	4 (4) 25.0%	0	0
Blood LDH increased	2 (2) 13.3%	1 (1) 6.3%	3 (4) 20.0%	2 (2) 13.3%
Insomnia	2 (2) 13.3%	2 (2) 12.5%	3 (3) 20.0%	3 (3) 20.0%

Data are presented as number of patients (number of events) and percentage of patients

*AE* adverse event, *ALT* alanine aminotransferase, *AST* aspartate aminotransferase, *LDH* lactate dehydrogenase

^a^Incidence ≥ 20% in any lusutrombopag group

^b^Coded by MedDRA terminology at the Preferred Term level

A total of 16 ADRs occurred in 11 of 61 patients (18.0%) in the safety analysis set: 10 ADRs in five of 15 patients (33.3%) in the 2-mg group, three ADRs in three of 16 patients (18.8%) in the 3-mg group, three ADRs in three of 15 patients (20.0%) in the 4-mg group, and no ADRs in the placebo group. These ADRs were positional vertigo, mesenteric vein thrombosis, malaise, pyrexia, hepatic dysfunction, portal vein thrombosis, elevated serum bilirubin, reduced serum fibrinogen, hypertension, elevated fibrin d-dimer and fibrin degradation products, lymphopenia, arthralgia, headache, and rash. These ADRs were not dose-dependent.

Serious AEs were reported in three patients (four events) in the 2-mg group, one patient (two events) in the 3-mg group, and one patient (one event) in the placebo group. Among these, two events in the 2-mg group and one event in the 3-mg group were related to hemorrhage. The patient with upper gastrointestinal hemorrhage in the 2-mg group died. None of the serious AEs were considered to have been caused by the study drug.

In the results of the blood coagulation and fibrinolysis tests in each lusutrombopag group, the mean activated partial thromboplastin time on Day 17 was slightly longer than that in the placebo group, but no trend of a dose-related increase was observed (Supplement S5). On Day 17, the mean values of fibrinogen, fibrinogen degradation products, and D-dimer seemingly increased from baseline in each group, including the placebo group. These changes were considered to be only a mild postoperative reaction caused by RFA independent of study drug effect.

A summary of the thrombosis- and bleeding-related AEs is shown in Table 3. Thrombosis-related AEs occurred in one of 15 patients (6.7%) in the 2-mg group, two of 15 patients (13.3%) in the 4-mg group, and one of 15 patients (6.7%) in the placebo group. None occurred in the 3-mg group. No dose-related increase in the incidence of thrombosis-related AEs was noted. In terms of severity, these events were moderate or severe, but not considered serious. PVT and superior mesenteric vein thrombosis that occurred in the patient in the 4-mg group were considered to have been related to the study drug. In these patients, the maximum platelet count was in the range of 57–127 × 10^3^/µL, and the platelet count immediately before the onset of the event was in the range of 37–91 × 10^3^/µL (Supplement S6). Bleeding-related AEs occurred in three of 15 patients (20.0%) in the 2-mg group, five of 16 patients (31.3%) in the 3-mg group, four of 15 patients (26.7%) in the 4-mg group, and eight of 15 patients (53.3%) in the placebo group (Table 3), which indicated that the bleeding-related AEs occurred more often in the placebo group than in the lusutrombopag groups. No dose-related increase in the incidence of bleeding-related AEs was noted.

**Table 3:** Thrombosis- and bleeding-related adverse events

	Lusutrombopag			Placebo *n* = 15
	2 mg *n* = 15	3 mg *n* = 16	4 mg *n* = 15	
Thrombosis-related adverse events^a^				
Patients with any thrombosis-related AE	1 (2) 6.7%	0	2 (2) 13.3%	1 (1) 6.7%
Mesenteric vein thrombosis	0	0	1 (1) 6.7%	1 (1) 6.7%
Portal vein thrombosis	1 (1) 6.7%	0	1 (1) 6.7%	0
Hepatic infarction	1 (1) 6.7%	0	0	0
Bleeding-related adverse events^a^				
Patients with any bleeding-related AE	3 (7) 20.0%	5 (8) 31.3%	4 (6) 26.7%	8 (15) 53.3%
Hematochezia	1 (1) 6.7%	0	1 (1) 6.7%	1 (1) 6.7%
Gingival bleeding	0	0	0	2 (2) 13.3%
Tongue hematoma	0	0	0	1 (1) 6.7%
Upper GI hemorrhage	1 (1) 6.7%	0	0	0
Hemorrhagic erosive gastritis	1 (1) 6.7%	0	0	0
Postprocedural hemorrhage	2 (2) 13.3%	0	3 (3) 20.0%	1 (1) 6.7%
Procedural hemorrhage	0	2 (3) 12.5%	0	1 (1) 6.7%
Incision site hemorrhage	0	1 (2) 6.3%	0	1 (1) 6.7%
Postprocedural hematoma	1 (1) 6.7%	0	0	1 (1) 6.7%
Contusion	0	0	0	1 (1) 6.7%
Hematuria	0	0	0	1 (1) 6.7%
Epistaxis	1 (1) 6.7%	1 (1) 6.3%	0	3 (4) 20.0%
Hemorrhage subcutaneous	0	2 (2) 12.5%	1 (1) 6.7%	1 (1) 6.7%
Purpura	0	0	1 (1) 6.7%	0

Data are presented as number of patients (number of events) and percentage of patients

Thrombosis-related AEs were defined as AEs belonging to the subqueries of “Embolic and thrombotic events, arterial (SMQ),” “Embolic and thrombotic events, venous (SMQ),” and “Embolic and thrombotic events, vessel type unspecified and mixed arterial and venous (SMQ)”

Bleeding-related AEs are defined as AEs belonging to the subquery of “Hemorrhage terms (excluding laboratory terms) (SMQ)”

*AE* adverse event, *GI* gastrointestinal

^a^Coded by Preferred Term

No clinically significant findings were noted in patient vital signs or electrocardiogram readings.

## Discussion

In the present study, the efficacy, safety, and pharmacokinetics of lusutrombopag were assessed at multiple oral doses of 2, 3, or 4 mg for up to 7 days for the pretreatment of RFA for primary hepatic carcinoma in patients with CLD and thrombocytopenia. Regarding efficacy, more than 80% of patients receiving lusutrombopag avoided platelet transfusion, and there was a modest dose-dependent increase in this endpoint as well as in the mean maximum platelet count that was reached. The number of days during which the criterion for being a responder was met tended to be lower with the 2-mg dose than with the 3- and 4-mg doses; thus, the 2-mg dose would afford clinicians less flexibility if a delay or repeat of the elective procedure was needed.

The ELEVATE study was the first multicenter, double-blind, randomized, placebo-controlled, phase 3 trial to evaluate the efficacy of a TPO-RA, eltrombopag 75 mg for 14 days, in increasing the platelet count and reducing the need for platelet transfusions in CLD patients with thrombocytopenia planned to undergo an elective invasive procedure [13]. The proportion of patients who did not require platelet transfusion was 72%. Unfortunately, the ELEVATE study was halted prematurely for safety concerns; although the platelet count-increasing effect of eltrombopag was significantly greater than that of placebo, eight patients had 10 thrombotic events (six patients experienced seven events in the eltrombopag group; two patients experienced three events in the placebo group).

Regarding the safety of lusutrombopag, we found that no dose-related AEs were observed, but two asymptomatic thrombotic events in the patients treated with 4 mg lusutrombopag were considered to have been related to the study drug even though no excessive platelet count rise was noted in these patients. In the ELEVATE study, among six patients in the eltrombopag group who experienced thrombotic events, the platelet count of five patients increased to more than 200 × 10^3^/µL, indicating that excessive increment of platelet count in CLD patients may lead to an increased risk of thrombotic events [13]. Only one subject in the present study who was treated with lusutrombopag 3 mg had a platelet count approaching this threshold, and the platelet count increased to 195 × 10^3^/μL, possibly because of the high variability in platelet count that was observed in this patient before lusutrombopag administration. It is important to weigh the risk of bleeding against the risk of excessively high platelet count, which could contribute to the development of PVT. One death occurred in the 2-mg group (upper gastrointestinal hemorrhage). The patient had a history of esophageal varices prior to entering the study; thus, the AE was attributed to variceal rupture, and a causal relationship to the study drug was ruled out. Among the doses examined in this study, lusutrombopag 3 mg QD for 7 days was considered the most appropriate, considering the balance between efficacy and safety.

The need for preoperative platelet transfusion prior to RFA was determined after assessment completion on Day 8 and immediately before RFA (within 2 days before the day of RFA). Preoperative platelet transfusion was performed only when the platelet count, obtained within the period of determination of the need for platelet transfusion, was < 50 × 10^3^/μL. If platelet counts were measured more than once within 2 days before the day of RFA and the values were ≥ 50 × 10^3^/μL at least once, preoperative platelet transfusion could not be performed. Although these criteria lack sufficient evidence with respect to CLD patients specifically, they are based on general guidelines in common use (Japanese guidelines for performance of blood transfusion and usage of blood products, available in Japanese at http://www.jrc.or.jp/vcms_lf/iyakuhin_benefit_guideline_sisin090805.pdf) and we believe that they are appropriate from the standpoint of evaluating the efficacy of lusutrombopag. Currently, there is no established consensus on the appropriate threshold for administering platelet transfusions in patients with CLD, and clinicians may rely on the general guidelines as described above.

Low plasma levels of antithrombin, protein C, and protein S by advanced liver cirrhosis were found to be associated with PVT development, but reduced portal vein flow velocity was the only variable independently associated with PVT development [14]. Previous reports showed that platelet count, albumin level, and decreased portal flow velocity were associated with the risk of thrombotic events after treatment with TPO-RAs in patients with liver diseases [15]. Among the blood coagulation and fibrinolysis factors tested in the present study, antithrombin III (%) and protein C activity (%) were seemingly lower, and von Willebrand factor activity (%) was seemingly higher when compared with the values in healthy subjects (79–121%, 64–146%, and 60–170%), confirming that CLD patients are at risk of developing PVT, especially after RFA.

In Europe, the Committee for Medicinal Products for Human Use investigated the thrombotic events that occurred in the ELEVATE study as part of the regulatory review on the eltrombopag marketing authorization for chronic immune thrombocytopenia. However, the committee could not identify a cause of the events or a causal relationship with eltrombopag because the study did not monitor pro- or anti-coagulant factors. Additionally, the study protocol was not specifically designed to assess a risk of PVT by abdominal diagnostic imaging [16].

Indeed, no well-controlled prospective study has been performed to assess the risk of PVT. For the clinical development of lusutrombopag for thrombocytopenia in CLD patients, we prospectively assessed the risk of PVT by the following means: (a) image assessments of the portal vein for all enrolled patients after invasive procedures, (b) a measure for preventing an excessive increase in the platelet count during the course of the study, and (c) successive monitoring of pro- and anti-coagulant factors.

The thrombotic events that occurred in the present study were all asymptomatic, and were all detected in image inspections after the invasive procedures. Furthermore, there was no definite tendency for a dose-related increment of thrombotic events. The present study is the first clinical study to show that the oral TPO-RA lusutrombopag could reduce the need for platelet transfusion in CLD patients undergoing invasive procedures while making a proactive assessment of the PVT risk.

The efficacy and safety in non-cancer CLD, and/or other procedures should be investigated further because the present study comprises a limited number of patients. Additionally, the sample size is too small to fully examine the safety profile, and any of the thrombotic events may have resulted from the RFA. The differences in patient background factors and the effects (number of patients with increased platelets, duration of platelet count increase, bleeding risk) of platelet transfusion on the efficacy and safety of lusutrombopag warrant further investigation in a large-scale study. Finally, the velocity of portal vein flow, a known predictor of PVT, was not evaluated in the present study because of both a lack of a suitable cutoff value for predicting increased risk and logistical difficulties in standardizing such measuring equipment across multiple study center sites.

In conclusion, lusutrombopag 3 mg QD for 7 days was found to be effective in increasing the platelet count sufficiently to avoid platelet transfusion while maintaining a favorable safety profile in terms of PVT incidence and risk of an excessive increase in the platelet count. Lusutrombopag demonstrated statistically significant reductions in platelet transfusions in CLD patients undergoing RFA. A dose-related increase in the maximum platelet count and duration of the maintenance of the increase in platelet count was noted. No trend of dose-related increase was noted for the incidence of adverse events. No significant safety concerns were raised with lusutrombopag at a dose of up to 4 mg vs placebo in thrombocytopenic patients with CLD. Lusutrombopag may be an alternative to platelet transfusion for elective invasive procedures in CLD patients.

Based on the results of this phase 2b study, a phase 3 confirmatory study (L-PLUS 1: JapicCTI-132323) including a wide variety of invasive procedures (not restricted to only RFA), was conducted in Japan, the results of which will be reported separately. Following positive results from this phase 2b study (JapicCTI-121944) and the phase 3 study (L-PLUS 1: JapicCTI-132323) conducted in Japan, a global phase 3 clinical study (L-PLUS 2: NCT02389621) was conducted.

## Electronic supplementary material

Below is the link to the electronic supplementary material.

Supplementary material 1 (PDF 68 kb)

Supplementary material 2 (PDF 62 kb)

Supplementary material 3 (PDF 188 kb)

Supplementary material 4 (PDF 36 kb)

Supplementary material 5 (PDF 70 kb)

Supplementary material 6 (PDF 61 kb)
