# Supplementary material for: A randomized controlled trial of lusutrombopag in Japanese patients with chronic liver disease undergoing radiofrequency ablation
Source: J Gastroenterol. 2018 Aug 13;54(2):171–81. doi: 10.1007/s00535-018-1499-2 (PMC6349796; doi:10.1007/s00535-018-1499-2)
Supplement: Supplementary file 1 — Supplementary material 1 (PDF 68 kb) [file 535_2018_1499_MOESM1_ESM.pdf]

Supplement S1. List of participating study centers

Hokkaido University Hospital; Sapporo Kosei General Hospital; Teine Keijinkai Hospital; Iwate Medical University Hospital; Saitama Medical University Hospital; Chiba University Hospital; Toho University Medical Center Omori Hospital; NTT Medical Center Tokyo; Tokyo Women's Medical University Hospital; Tokyo Medical University Hospital; Kanto Central Hospital of the Mutual Aid Association of Public School Teachers; Mitsui Memorial Hospital; The University of Tokyo Hospital; Tokyo Metropolitan Cancer and Infectious Diseases Center Komagome Hospital; Toranomon Hospital; Musashino Red Cross Hospital; Yokohama City University Medical Center; Toranomon Branch Hospital; Kitasato University East Hospital; Saiseikai Niigata Daini Hospital; Kanazawa University Hospital; Gifu Municipal Hospital; Ogaki Municipal Hospital; Mie University Hospital; University Hospital, Kyoto Prefectural University of Medicine; Kyoto University Hospital; Osaka City University Hospital; Osaka Red Cross Hospital; Osaka Medical Center for Cancer and Cardiovascular Diseases; Osaka University Hospital; Ikeda City Hospital; National Hospital Organization Osaka Minami Medical Center; Kishiwada City Hospital; Osaka Rosai Hospital; Kansai Medical University Takii Hospital; Kindai University Hospital; Faculty of Medicine, National Hospital Organization Osaka Medical Center; Kansai Rosai Hospital; Meiwa Hospital; Hyogo College of Medicine Hospital; Hyogo Prefectural Nishinomiya Hospital; Nishi-Kobe Medical Center; Nara Medical University Hospital; Wakayama Medical University Hospital; Saiseikai Wakayama Hospital; Okayama Citizens' Hospital; Okayama University Hospital; Kurashiki Central Hospital; Chugoku Rosai Hospital; Hiroshima City Hiroshima Citizens Hospital; Tokushima Prefectural Central Hospital; Kagawa Prefectural Central Hospital; Ehime Prefectural Central Hospital; Matsuyama Red Cross Hospital; Ehime University Hospital; Iizuka Hospital; Steel Memorial Yawata Hospital; Kurume University Hospital; Fukuoka University Hospital; National Hospital Organization Kyushu Medical Center; Saga Prefectural Hospital Koseikan; Kumamoto University Hospital; and Oita University Hospital
