# Supplementary material for: A randomized controlled trial of lusutrombopag in Japanese patients with chronic liver disease undergoing radiofrequency ablation
Source: J Gastroenterol. 2018 Aug 13;54(2):171–81. doi: 10.1007/s00535-018-1499-2 (PMC6349796; doi:10.1007/s00535-018-1499-2)
Supplement: Supplementary file 2 — Supplementary material 2 (PDF 62 kb) [file 535_2018_1499_MOESM2_ESM.pdf]

Supplement S2. **List of prohibited therapies during the study period**

Platelet preparations (except in patients who received platelet transfusion once prior to percutaneous liver ablation based on the determination of the need for preoperative platelet transfusion and for use as rescue therapy); blood preparations except for platelet preparation, red blood cell preparation, and albumin preparation; anticancer drugs (except for transcatheter hepatic arterial chemoembolization and lipiodolization after completion of assessment on Day 8); interferon preparations; macrophage colony-stimulating factor products; granulocyte colony-stimulating factor products; erythropoietin; TPO receptor agonists; antithrombotics (heparin, aspirin, dipyridamole, ticlopidine, urokinase, among others) except for use as rescue therapy; hemostatic agents except for topical use and for use as rescue therapy; other investigational products; thoracotomy; laparotomy; splenectomy; partial splenic embolization; hepatectomy; liver transplantation; percutaneous ethanol injection therapy; transcatheter arterial infusion (except for lipiodolization); radiotherapy; and endoscopic injection sclerotherapy.
