# Supplementary figures and images for: A randomized controlled trial of lusutrombopag in Japanese patients with chronic liver disease undergoing radiofrequency ablation
Source: J Gastroenterol. 2018 Aug 13;54(2):171–81. doi: 10.1007/s00535-018-1499-2 (PMC6349796; doi:10.1007/s00535-018-1499-2)

## Supplement S3. Patient disposition

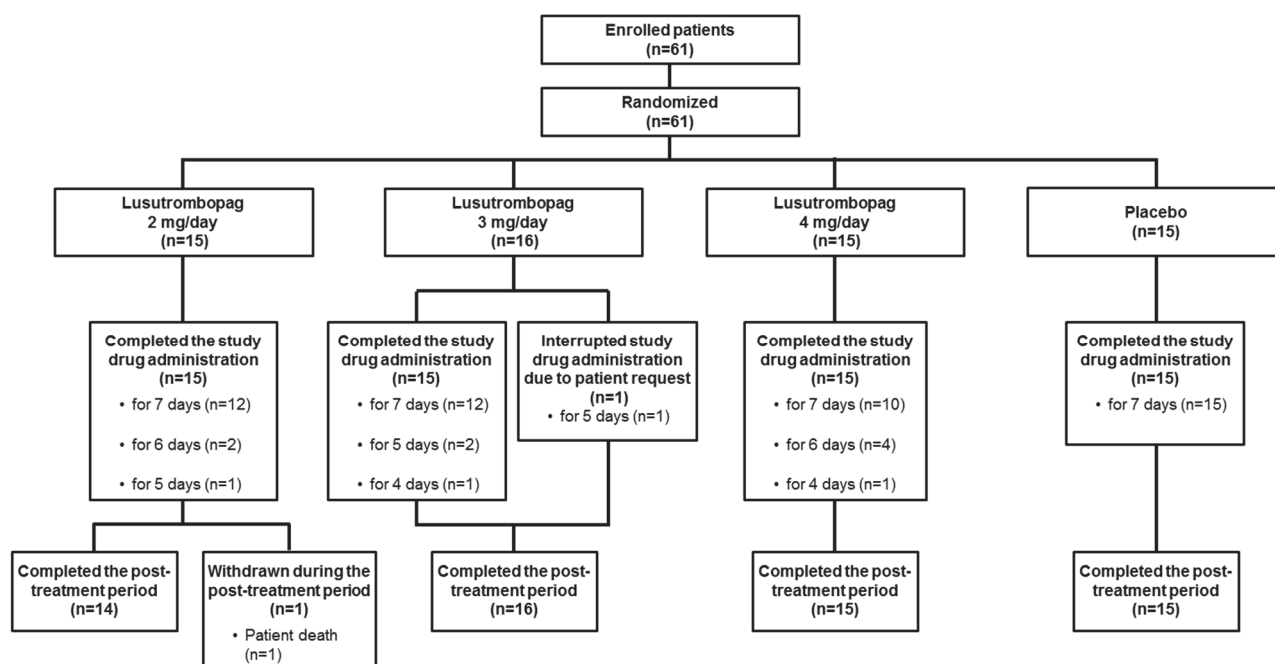

Supplement: Supplementary file 3 — Supplementary material 3 (PDF 188 kb) [file 535_2018_1499_MOESM3_ESM.pdf]
