# Supplementary material for: A randomized controlled trial of lusutrombopag in Japanese patients with chronic liver disease undergoing radiofrequency ablation
Source: J Gastroenterol. 2018 Aug 13;54(2):171–81. doi: 10.1007/s00535-018-1499-2 (PMC6349796; doi:10.1007/s00535-018-1499-2)
Supplement: Supplementary file 4 — Supplementary material 4 (PDF 36 kb) [file 535_2018_1499_MOESM4_ESM.pdf]

Supplement S4. Details of platelet transfusions

	Lusutrombopag				
	**2 mg n = 15**	**3 mg n = 16**	**4 mg n = 15**	**Placebo n = 15**	**Overall N = 61**
Patients with platelet transfusion	3 (20.0)	3 (18.8)	4 (26.7)	12 (80.0)	22 (36.1)
Reason for platelet transfusion:					
Platelet count <50 $\times 10^3/\mu L$ before RFA	2 (13.3)	3 (18.8)	3 (20.0)	12 (80.0)	20 (32.8)
Adverse events related to bleeding	0	1 (6.3)	0	0	1 (1.6)
Other	1 (6.7)[a]	1 (6.3)[b]	1 (6.7)[c]	1 (6.7)[d]	4 (6.6)
Number of platelet transfusions per patient					
1	2 (13.3)	2 (12.5)	4 (26.7)	8 (53.3)	16 (26.2)
2	1 (6.7)	0	0	3 (20.0)	4 (6.6)
3	0	1 (6.3)	0	1 (6.7)	2 (3.3)
Mean dose (JP unit[e]), mean ± SD	10.0 ± 0.0	17.8 ± 6.9	12.5 ± 5.0	11.7 ± 2.5	12.4 ± 4.1

Data are presented as n (%) unless otherwise stated.

[a]Received platelet transfusion to ensure safety, because platelet count decreased to 45 $\times 10^3/\mu L$ on the day of surgery.

[b]Received second platelet transfusion prior to permanent pacemaker indwelling (first platelet transfusion was performed because platelet count was <50 $\times 10^3/\mu L$ before percutaneous liver ablation).

[c]Received platelet transfusion after percutaneous liver ablation because platelet count was <50 $\times 10^3/\mu L$ on Day 21.

[d]Received second transfusion after percutaneous liver ablation (first platelet transfusion was performed because platelet count was <50 $\times 10^3/\mu L$ before percutaneous liver ablation).

[e]1 JP unit contains ~2 $\times 10^{10}$ platelets.

Abbreviations: RFA, radiofrequency ablation; SD standard deviation
