# Supplementary material for: A randomized controlled trial of lusutrombopag in Japanese patients with chronic liver disease undergoing radiofrequency ablation
Source: J Gastroenterol. 2018 Aug 13;54(2):171–81. doi: 10.1007/s00535-018-1499-2 (PMC6349796; doi:10.1007/s00535-018-1499-2)
Supplement: Supplementary file 5 — Supplementary material 5 (PDF 70 kb) [file 535_2018_1499_MOESM5_ESM.pdf]

Supplement S5. Change in blood coagulation/fibrinolysis parameters

	Lusutrombopag			
	**2 mg n = 15**	**3 mg n = 16**	**4 mg n = 15**	**Placebo n = 15**
PT INR				
Baseline	1.31 ± 0.11	1.29 ± 0.13	1.23 ± 0.10	1.27 ± 0.10
Day 8	1.30 ± 0.12	1.29 ± 0.13	1.28 ± 0.09	1.27 ± 0.09
Day 17	1.35 ± 0.11	1.31 ± 0.14	1.36 ± 0.33	1.30 ± 0.10
Day 35	1.30 ± 0.09	1.25 ± 0.13	1.33 ± 0.18	1.28 ± 0.09
Activated partial thromboplastin time (s)				
Baseline	34.61 ± 3.75	33.38 ± 4.21	34.52 ± 4.45	34.16 ± 3.78
Day 8	34.59 ± 4.54	33.58 ± 3.12	36.51 ± 3.91	34.26 ± 4.06
Day 17	36.07 ± 4.45	34.80 ± 5.96	36.55 ± 6.57	33.17 ± 3.55
Day 35	34.39 ± 4.66	32.71 ± 4.64	34.69 ± 4.48	34.28 ± 4.14
Fibrinogen (mg/dL)				
Baseline	165.5 ± 38.0	187.8 ± 71.1	163.4 ± 37.2	185.8 ± 45.4
Day 8	175.9 ± 45.5	172.5 ± 39.2	165.6 ± 46.2	184.9 ± 44.3
Day 17	235.5 ± 91.0	233.6 ± 90.0	218.9 ± 81.4	207.8 ± 76.2
Day 35	189.4 ± 57.6	188.0 ± 61.1	172.7 ± 45.9	179.7 ± 37.4
Fibrinogen degradation product (μg/mL)				
Baseline	3.1 ± 1.9	2.9 ± 1.6	2.8 ± 1.5	3.7 ± 3.4
Day 8	3.3 ± 1.4	2.3 ± 0.8	3.2 ± 2.1	3.1 ± 2.5
Day 17	7.5 ± 6.7	6.3 ± 2.5	4.4 ± 2.7	14.3 ± 20.4
Day 35	5.6 ± 5.6	3.5 ± 1.6	3.3 ± 1.8	4.1 ± 2.8
Antithrombin III (%)				
Baseline	59.8 ± 15.6	62.2 ± 21.5	56.1 ± 14.2	60.5 ± 22.7
Day 8	60.9 ± 13.2	59.5 ± 21.3	49.8 ± 14.3	57.8 ± 20.8
Day 17	55.8 ± 11.2	60.2 ± 23.2	55.4 ± 16.5	57.1 ± 20.0
Day 35	61.1 ± 13.9	62.5 ± 20.3	56.1 ± 14.7	56.7 ± 20.0
D-dimer (μg/mL)				
Baseline	0.865 ± 0.832	0.751 ± 0.698	0.577 ± 0.603	1.083 ± 1.327
Day 8	0.915 ± 0.675	0.505 ± 0.444	0.778 ± 0.847	1.068 ± 1.327
Day 17	2.143 ± 1.707	1.862 ± 0.921	1.551 ± 1.140	5.196 ± 7.884
Day 35	1.429 ± 1.067	0.963 ± 0.726	0.812 ± 0.709	1.275 ± 1.286

Data are presented as mean ± SD.

Abbreviation: PT INR, international normalized ratio of prothrombin time
