# Supplementary material for: A randomized controlled trial of lusutrombopag in Japanese patients with chronic liver disease undergoing radiofrequency ablation
Source: J Gastroenterol. 2018 Aug 13;54(2):171–81. doi: 10.1007/s00535-018-1499-2 (PMC6349796; doi:10.1007/s00535-018-1499-2)
Supplement: Supplementary file 6 — Supplementary material 6 (PDF 61 kb) [file 535_2018_1499_MOESM6_ESM.pdf]

Supplement S6. Summary of thrombus-related adverse events

Sex/Age	Group	Platelet transfusion	Baseline platelet count ($\times10^3/\mu L$)	Maximum platelet count (study day) / platelet count immediately before the onset (study day) ($\times10^3/\mu L$)	Type of procedure (study day)	Name of event	Relationship	Duration of event[a]	Outcome
M/70	Placebo	Yes	47	62 (Day 17) / 62 (Day 17)	RFA (Day 9, 16, 23)	Superior mesenteric vein thrombosis	Not related	Day 19–121	Recovered
M/63	2 mg/day	Yes	43	57 (Day 27) / 37 (Day 17)	RFA (Day 13)	PVT	Not related	Day 18–34	Not recovered[d]
F/81	4 mg/day	No	43	105 (Day 19) / 91 (Day 14)	TACE[b] (Day 8) RFA (Day 11)	Superior mesenteric vein thrombosis	Possibly related	Day 14–42	Recovered[e]
F/77	4 mg/day	No	47	127 (Day 11, 13) / 85 (Day 18)	RFA (Day 13) PEIT[c] (Day 13)	PVT	Possibly related	Day 18–49	Not recovered[f]

[a]The day of the initial dose of study drug is Day 1.

[b, c]Procedures performed in combination with RFA (primary invasive procedure).

[d]Patient was treated with danaparoid sodium for PVT, but did not recover from PVT at end of the post-treatment period. However, as general status was resolved, study follow-up was switched to usual care. The portal vein flow had been detected at onset.

[e]Patient treated with heparin and warfarin in therapy for PVT.

[f]Patient did not recover from PVT at end of the post-treatment period. However, as the flow of portal vein was stable without symptoms, study follow-up was switched to usual care.

Abbreviations: RFA, radiofrequency ablation; PEIT, percutaneous ethanol injection therapy; PVT, portal vein thrombosis; TACE, transcatheter arterial chemoembolization
